# Using CTSA infrastructure to address health disparities in New York City communities: Barriers and facilitators to implementing a free health screening program

**DOI:** 10.1017/cts.2025.46

**Published:** 2025-03-06

**Authors:** Jifeng (Jeff) Zhu, Michael E. Bales, Christine A. Ganzer, Farid Aboharb, Allegra Keeler, Krista A. Ryon, Ana C. Benitez, Brett J. Ehrmann, Julianne Imperato-McGinley

**Affiliations:** 1 Weill Cornell Clinical and Translational Science Center, New York, NY, USA; 2 Hunter-Bellevue School of Nursing, School of Health Professions, Hunter College, CUNY, New York, NY, USA; 3 Tri-Institutional MD-PhD Program, Weill Cornell Medicine, Rockefeller University, Memorial Sloan Kettering Cancer Center, New York, NY, USA; 4 School of Medicine, New York Medical College, Valhalla, NY, USA; 5 Department of Physiology and Biophysics, Weill Cornell Medicine, New York, NY, USA; 6 Division of Primary Care of the Weill Cornell Physician Organization, Weill Cornell Medicine, New York, NY, USA

**Keywords:** Community health partnerships, diseases, New England, health disparities, community-based participatory research, cardiovascular disease, diabetes

## Abstract

The Weill Cornell Heart to Heart Community Outreach Campaign (H2H) is a free outreach program that provides mobile health screenings. The program brings medical and nursing faculty and students to the underserved, uninsured communities of New York City. Participants are screened for diabetes and heart disease risk factors through onsite exams, including point of care blood tests. If an abnormality is found, they receive a medical consultation to offer personalized advice and referrals to free/low-cost clinics when needed. The goal is to help underserved individuals understand their cardiometabolic health and to promote early intervention. This article describes the development of the program, including factors that were essential to the collaboration, challenges faced, barriers to implementation, and its evolution throughout the first 12 years. The program has benefited from strong foundational program leadership, effective inter-institutional collaboration, and maintaining community trust.

## Introduction

**Figure 1.**
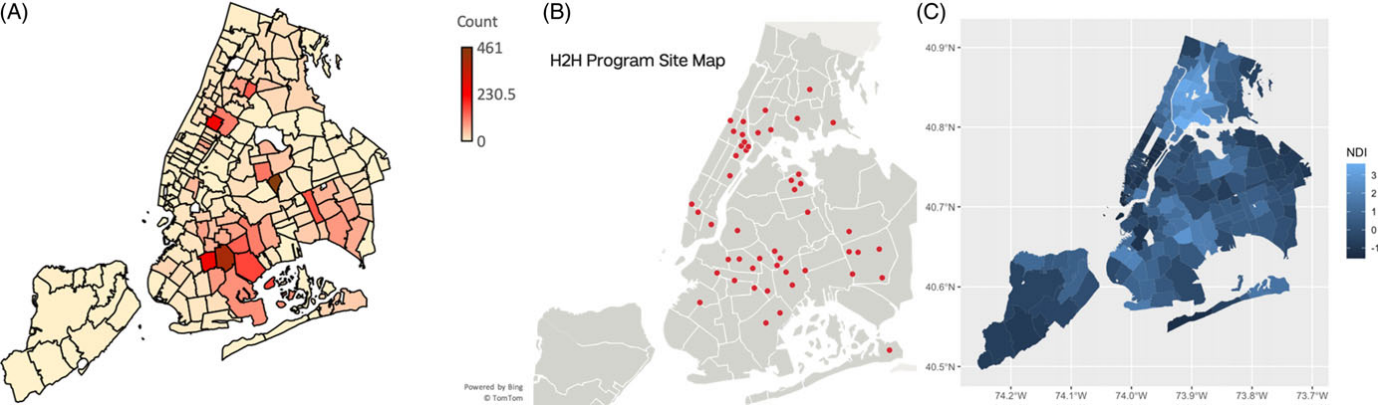
**(A**) a thematic map showing the geographical distribution of H2H participants throughout the five boroughs of NYC. (**B)** A map of sites participating in the heart to heart community outreach program (see acknowledgments for a list of sites). (**C)** A map of the neighborhood deprivation index for NYC (light blue indicates underserved neighborhoods). This figure first appeared in the translational science case study describing the H2H program [1].

The Heart-to-Heart Community Outreach Campaign (H2H) [1] is a mobile community outreach program, free to participants, that provides screenings for diabetes and other cardiovascular disease (CVD) risk factors to underserved and minority communities in New York City (NYC) (see Figure 1, below). The screening events are hosted in partnership with faith-based institutions. Participants are asked to provide a medical history, complete a survey, and receive counseling by clinicians with referrals for follow-up care when needed. The population served is disproportionately non-white, and uninsured, with many low-income and underserved individuals. The program found that over half of participants screened had an HbA1c in the prediabetic or diabetic range. The prevalence of diabetes (both diagnosed and undiagnosed), as well as hypertension and obesity, is significantly higher than those of adults in New York City as a whole. These findings are described in detail in a Translational Science Case Study, which also describes the clinical tests performed, frequency of events, and annual participation [1].

This article describes the roles of key stakeholders in the program’s growth and development, factors that are essential to facilitate collaboration, and challenges and barriers to implementation. The program has stimulated significant community engagement in the translational research process at our Clinical and Translational Science Center (CTSC). We believe this program will be of use to others planning community-partnered research initiatives.

### The early years: identifying the target community and establishing the community partnership

H2H was initiated by two MD/PhD students at WCM to combat CVD in underserved NYC populations [2]. Inspired by their experience as student co-directors of the student-run, free Weill Cornell Community Clinic, they wanted to “bring the clinic to the community” and approached the principal investigator of our CTSC hub who set the project in motion. With help from CTSC External Advisory Board member Reverend Patrick O’Connor, a pilot event was organized at his church, which serves predominantly low-income African American and Hispanic neighborhoods.

The program gained momentum when it caught the attention of a state senator’s chief of staff at the first event; additional events were scheduled in collaboration with the senator’s office. Simultaneously, CTSC staff worked with the CTSC Community Advisory Board to extend the program’s reach.

Word of mouth spread to other faith-based organizations and proved an effective method for establishing new partnerships. Successful early events led to many requests from other faith-based organizations by the third year. Noting the impact and collaborative process of H2H screening events, the number of requests for events steadily grew to dozens per year.

The WCM Institutional Review Board approved the H2H study in 2010 for generally healthy individuals over 18, and, as mentioned earlier, re-classified it in 2023 as quality assurance/quality improvement (QA/QI). By removing the program from human subject research regulations, the informed consent process is now much simpler, and data from participants who had previously been deterred by the informed consent process can now be collected and aggregated anonymously. This is particularly pertinent for inclusion of data from the large number of non-English speaking participants who previously declined to enroll in the research study portion of the program, but still received a free screening.

### Building trust with community stakeholders and expanding community involvement in the translational research process

With the support of community partners and clergy members, H2H defined its goal of reaching out to the underserved by holding health screening events among low-income minority groups in NYC. Community leaders were essential in advocating for neighborhood events, fostering trust, and determining the screenings offered. Over time, the program continued to gain attention and recognition, and just three years after H2H was initiated, the number of requests for events began to exceed the program’s capacity.

A major CTSC goal for this program was to develop long-term partnerships with underserved communities across NYC. There is an increase in trust among community members after multiple consecutive annual events at a site, and these annual events have likely helped assuage participants that they are not being taken advantage of by the medical establishment (a perception that has been well-documented in research involving underserved communities) [3,4]. This increased trust has made community members willing to participate in the CTSC’s Community Advisory Board (CAB), assist in CTSC-supported studies, and serve as collaborators on research studies led by CTSC principal investigators. In addition, the program serves as a crucial platform to support researchers, providing a mechanism to not only recruit individuals traditionally underrepresented in research for studies but also obtain valuable feedback from members of these communities.

Over the years, many of the CTSC’s CAB members have come from partners of the H2H program. In one of the CTSC’s key annual processes, CAB members review and score all our pilot award applications from a community perspective. This time-intensive process requires numerous community members willing to devote hours of time each year, making the good will and trust developed by the H2H program essential to the continuation of this initiative. Thus, H2H is a bridge, allowing community engagement throughout the translational research process.

At least two unanticipated community-level effects have emerged from H2H. First, despite the program organizers’ emphasis to the contrary, some participants have expressed that the program serves as their primary source of healthcare and is regarded by some community members as an annual tradition. While H2H is not intended for this purpose, some communities have large numbers of undocumented immigrants who have difficulty obtaining healthcare and health insurance.

### Sharing the results of the research with the community

The CTSC hub provides updates regularly to the CTSC External Advisory Board, as well as its Community Advisory Board. Updates on the program have also been shared nationally at meetings of sub-committees of the National Center for Advancing Translational Sciences (NCATS) [5]. Additionally, medical students, who develop and propose the research questions for the Program, have presented the H2H Program at several conferences and events. Members of the Community team at the CTSC present about the program on a regular basis at internal meetings and educational lectures at partner institutions such as Hunter College and Cornell University. In total, the program has been presented by CTSC staff and program leadership at over 50 meetings ranging from the institutional level to the national level. In addition, five papers involving work accomplished through the H2H program and its infrastructure have been published over the years [1,6–9], with CTSC staff being lead or co-authors on almost all. In addition, dozens of other CTSC supported studies have used H2H events to help recruit participants, typically through distribution of flyers by CTSC staff or study team members at H2H events.

### Supporting multidisciplinary collaboration

Establishing and maintaining trust with community partners through H2H has also led to increased participation among community members in other CTSC-related research initiatives. As a result, H2H has also been a hub for clinical innovation and multidisciplinary collaboration. Over the years, representatives of several CTSC-supported research studies have attended H2H events to recruit participants and complete study activities on site. One such study was a 2018 device trial with startup company VitaScan for a point-of-care device they developed to test iron levels in blood [10]. In another study, two MD/PhD students designed a 3D-printed iPhone adapter, a makeshift fundus camera for retinal imaging [7]. They secured a CTSC pilot grant to trial this device and introduce ophthalmology screenings to the H2H program, which continue to this day. Currently, we are supporting a device trial to test a magnet-based blood sample processing device designed to allow individuals to perform their own blood draws and processing at home. The CTSC has also used the program to support a variety of other studies with recruitment and enrollment of participants at H2H events (with participants enrolled by either study or CTSC staff). Examples include an Alzheimer’s study [6], a study evaluating food security survey interpretation in under-served populations [8], and the “All of Us” Research Program [11]. H2H program staff have also distributed flyers for numerous research studies. Overall, H2H serves as a trusted community partnership that facilitates connections between the medical establishment and underserved communities across NYC, contributing to involvement and awareness of research studies and trials.

The CTSC encourages collaboration with our institutional partners for the H2H initiative, especially when they provide resources, education, and contribute to a comprehensive screening for participants. In 2016, the H2H Medical Director learned that the leadership of NewYork-Presbyterian Hospital’s Dietetic Internship program sought to provide their interns with community health experience, leading to the addition of a nutrition counseling component. At many events, participants now receive culturally and medically tailored nutrition handouts, including modified recipes for cultural staples, and nutrition consultations from dietetic interns overseen by their licensed instructors. Recently, the CTSC collaborated with the Meyer Cancer Center [12] at Weill Cornell Medicine to add lung cancer, colon cancer, and prostate cancer screenings to some H2H events. In 2023, several Hunter undergraduate students interested in Social Work compiled and verified a database of low cost/free clinics in NYC to which to refer participants.

### Factors that were essential to the collaboration (facilitators)

**Figure 2.**
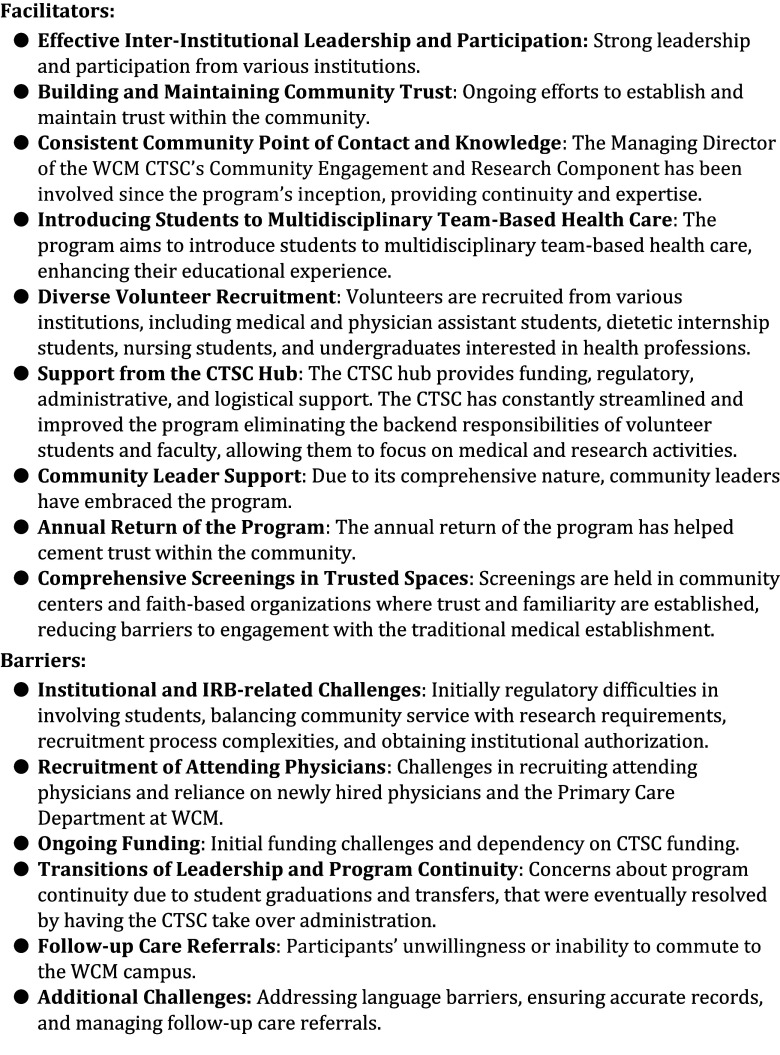
Summary of facilitators and barriers to ongoing implementation of the heart to heart program.

Facilitators that have been essential to the collaboration (Figure 2) include effective inter-institutional leadership and participation and building and maintaining community trust.

### Role of the CTSC in program success

The CTSC spends about $20K per year on test kits and supplies for the program, as well as about $10K per year for school bus and taxi rides to transport volunteers from the WCM campus to community sites hosting the screening events. The CTSC also provides an in-kind contribution of community engagement and research component staff toward this project. This includes four research assistants, a community network manager, and a managing director. The community team coordinates all Institutional Review Board (IRB) and legal/regulatory oversight for the program, plans the events with community partners, and handles all purchasing and logistics for the program. The CTSC also provides RedCAP [13] support for the project.

A major issue for many student-initiated efforts is the loss of institutional knowledge and expertise with student turnover. This is resolved for H2H by having a consistent point of contact and dedicated staff support through the CTSC’s Community Engagement and Research Component. The Managing Director of the component (J.Z.) has been involved since the program’s inception, serving as a consistent community point of contact and institutional knowledge over the years. Furthermore, having the CTSC’s professional team administer the program has allowed constant refinement and improvements to program processes that have resulted in a seamless experience for student and faculty volunteers. The CTSC team handles all backend activities, including maintaining community partnerships and planning events, and students are provided transportation to and from events via a rented bus. As a result, students and faculty involved can dedicate all their effort to the health screenings and to research they may be conducting.

Volunteers are recruited from WCM, NewYork-Presbyterian Hospital, and Hunter College (HC). WCM volunteers include medical and physician assistant students, NYP volunteers, and dietetic internship students. HC volunteers include nursing students and undergraduates from the college interested in exploring the health professions. In addition, the program recruits licensed medical professionals (physicians, nurse practitioners, and physician assistants) from WCM and HC to conduct educational counseling based on current guidelines [14–16]. A specific aim of the program is to introduce students to the concept of multidisciplinary team-based health care, and the presence of diverse students and faculty from many facets of healthcare has allowed students to be exposed to this early in their healthcare education.

During the first few years, the role of the CTSC hub was to provide funding and regulatory, administrative, and logistical support. With expansion, the CTSC hub handled most of the program’s organization, while also providing extensive resources and support for volunteer faculty/students and CTSC supported PI’s interested in conducting research with H2H program participants [6–11,17]. Support ranged from study design and regulatory approval assistance, to study recruitment, sample processing, data analysis, and manuscript drafting. A critical contribution of the CTSC community team was working with research teams to ensure that the study design and goals of the program were feasible given the population and permitted medical activities at H2H events.

Community leaders embraced the H2H program because it was the only comprehensive program most had encountered. While some programs offered singular point-of-care testing (lipid, blood pressure, mammograms, etc.), none were integrated into a comprehensive program that provided multiple tests, including referrals to follow-up care, referrals to free insurance enrollment assistance, and, most importantly, medical consultations onsite. The annual return of the program further cemented this trust. Comprehensive screenings are held in spaces provided by community centers and faith-based organizations where participant familiarity and trust are firmly established, thereby decreasing the barriers (real and perceived) to engaging with the traditional medical establishment.

### Challenges and barriers to implementation

Several key challenges were encountered when developing the H2H Program, including institutional hurdles before launching and recruitment of attending physicians.

### Institutional and IRB-related challenges

A significant challenge was how to involve students across our CTSA consortium. Organizers reasoned that the number of students participating (hundreds of students from WCM, NYP, and Hunter College) would be both large and frequently changing, making their addition to the IRB protocol impractical. The IRB eventually agreed that any healthcare students whose role was limited to volunteering at the medical screening events would not need to be listed on the IRB protocol; however, a record of those involved was requested. The second main institutional hurdle was balancing the program’s community service goals with the strict requirements of a public health research initiative, resulting in two challenges – one pertaining to the consent of participants and the other to institutional risk and insurance.

For the program’s first five years, to help mitigate the complexities of the recruitment process, participants were recruited anonymously. By keeping participants anonymous, the IRB initially determined that verbal consent would be sufficient, allowing the program to obtain consent from many participants (over 100 in some cases) in a very short period (on the order of an hour or two). However, this precluded the possibility of following up with participants to determine outcomes, severely hampering research efforts. As a result, the program was shifted to written consent in 2016 requiring a large number of CTSC staff at each event to handle written consent. The program resolved this issue by working with the IRB to reclassify the program’s survey as a quality assurance/quality improvement (QA/QI) project in 2023 as previously mentioned.

An additional challenge arose from medical care provided off-campus at locations unaffiliated with our medical college or hospital network. In a series of meetings involving H2H leadership, the leadership of the IRB, and the risk management office, it was ultimately determined that if an attending physician affiliated with the hospital network were present at all events to oversee medical activities, insurance would cover medical activities and student volunteers.

Additionally, the IRB and risk management required that communications with participants be limited to identifying likely cases of chronic disease, and that participants be referred for follow-up care to make an official diagnosis. Overall, obtaining authorization from relevant institutional offices required substantial effort during program development and continuous collaboration over the years to approve changes and additional screenings.

### Recruitment of attending physicians

The program has been fortunate to have the support of several highly dedicated medical faculty volunteers devoting their time and effort. A key strategy employed by program leadership has been to obtain a list of new attending physicians annually and to reach out to them by email. Organizers have found that newly hired attending physicians building their practices may have more interest in volunteering on weekends. The program has received strong support from the Primary Care Department at WCM, with most medical directors and volunteer physicians recruited from this group. Another successful strategy has been through medical students who identify prospective faculty volunteers through their coursework. In 2024, the CTSC began to offer a small stipend ($500) to acknowledge volunteer physicians for their time. To date, the volunteer physicians have declined this stipend and asked that the money be spent on the program. We will continue to monitor this stipend offer and determine best steps moving forward.

One of the key barriers to involving communities across the entire research pipeline is finding community leaders willing to contribute their time and expertise. This was an issue for H2H for the first couple of years before trust was established and spread via word of mouth.

### Ongoing funding

The program was initially funded through a two-year pilot award from the CTSC hub, followed by a grant from the AstraZeneca Foundation for two years. However, for most of its existence, the CTSC has directly funded the project through the community engagement and research component. Program leadership has reached out to several foundations over the years and has learned that while it is not uncommon for foundations to provide community engagement programs with start-up funding to support the first year or two, identifying foundation support for long-term funding is challenging. As a result, the program depends upon CTSC funding and continued grant renewal.

### Transitions of leadership and program continuity

As students eventually graduate, transfer schools, or leave for other reasons, program continuity is often a concern in student-run projects. While H2H founders were MD/PhD students who were present for many years, organizers realized early on that continuity was needed. To address these issues, the program shifted all administrative, regulatory, and logistical duties to the CTSC as previously mentioned. For Hunter nursing students’ participation, the program has been integrated into their curriculum, providing an opportunity to fulfill service-learning community requirements and thereby ensuring a continuous pool of volunteers. Hunter College undergraduate students comprise the largest group of volunteers. The program works closely with the pre-health careers club at Hunter College, a CTSC partner institution, which has hundreds of students interested in volunteer opportunities in the healthcare field. To manage this pool of volunteers, there are two Hunter undergraduate volunteer leaders who help recruit, train, and oversee the volunteers at events.

### Additional challenges

Two significant changes were made to the program after the first event. First, Spanish-speaking staff and volunteers were recruited to address the large non-English speaking Hispanic population in NYC. Second, to prevent participant confusion and ensure accurate records, undergraduate student volunteers were added to escort participants through the screening process and administer study surveys.

There were also two activities that organizers anticipated would be challenging for the long term but did not turn out to be: identifying community partners and recruiting student volunteers. As mentioned earlier, driven by the positive community response for annual returns, screening requests were abundant by the third and fourth years. As for recruitment of student volunteers, organizers initially anticipated challenges, as events require dozens of student volunteers. Although it is more difficult to find student volunteers at specific times of the year (the month of graduation, the summer, and holidays), there has never been a shortage. It has not been uncommon to receive requests from students who learned about the opportunity from students who attended and volunteered.

A final challenge has been referring participants to follow-up care if required. The direct referral of the program is to the student-run free clinic at WCM and not all participants are willing or able to commute to the WCM campus. Traditionally, we have provided participants with a list of free or low-cost clinics from the NYC Department of Health and Mental Hygiene; however, this list can quickly become outdated. In 2023, Hunter undergraduate students interested in Social Work compiled a comprehensive list of free/low-cost clinics in NYC and reached out to each to confirm details. Using this information, a REDCap [13] survey was developed to identify the appropriate clinic for participants based on address, insurance/immigration status, and other factors. A dedicated group of Hunter undergraduates keeps this database up to date and attends events to connect participants to follow-up care.

## Conclusion

Effective inter-institutional leadership, funding, and community trust-building have helped organizers to overcome recruitment, regulatory, and other challenges. The Heart to Heart program has grown into a robust and effective program that has provided screenings for diabetes and other CVD risk factors to NYC’s underserved and minority communities, while also bolstering community engagement within our CTSC. It is hoped that the information in this article may be useful to those working to develop similar community-based initiatives.
